# Morphological and biomechanical characterization of immature and mature nasoseptal cartilage

**DOI:** 10.1038/s41598-019-48578-3

**Published:** 2019-08-28

**Authors:** Zita M. Jessop, Yadan Zhang, Irina Simoes, Ayesha Al-Sabah, Nafiseh Badiei, Salvatore A. Gazze, Lewis Francis, Iain S. Whitaker

**Affiliations:** 1 Reconstructive Surgery and Regenerative Medicine Research Group, Swansea University Medical School, Swansea, UK; 2 The Welsh Centre for Burns and Plastic Surgery, Morriston Hospital, Swansea, UK; 3 Centre for NanoHealth, Institute of Life Sciences, Swansea University, Swansea, UK

**Keywords:** Cartilage development, Atomic force microscopy, Cartilage

## Abstract

Nasoseptal cartilage has been assumed to be isotropic, unlike the well-defined zonal organization of articular cartilage attributed to postnatal biomechanical loading. We know from clinical experience that malrotation of surgical nasoseptal cartilage grafts can lead to increased graft absorption. Other studies have also suggested directionally dependent compressive stiffness suggesting anisotropy, but morphological investigations are lacking. This study characterizes immature and mature native bovine nasoseptal cartilage using a combination of immunohistochemistry, biomechanical testing and structural imaging. Our findings indicate that there is extensive postnatal synthesis and reorganization of the extracellular matrix in bovine nasoseptal cartilage, independent of joint loading forces responsible for articular cartilage anisotropy. Immature nasoseptal cartilage is more cellular and homogenous compared to the zonal organization of cells and extracellular matrix of mature cartilage. Mature samples also exhibited greater glycosaminoglycan content and type II collagen fibre alignment compared to immature cartilage and this correlates with greater compressive stiffness. Engineered neocartilage often consists of immature, isotropic, homogenous tissue that is unable to meet the functional and mechanical demands when implanted into the native environment. This study demonstrates the importance of anisotropy on biomechanical tissue strength to guide future cartilage tissue engineering strategies for surgical reconstruction.

## Introduction

Autologous cartilage grafts are used widely in reconstructive surgery of the ear, nose and airway following defects from trauma, cancer resection and congenital conditions^1,2^. However, complications arising from the cartilage donor site, most commonly costochondral^3,4^, as well as the converging developments in cell biology and biofabrication, have increased interest in the potential to engineer cartilage for reconstruction^5^. In order to create durable tissue, it is important to first understand its native macro, micro and nanoarchitecture^6^. Being avascular, aneural and immune-privileged, means that cartilage is perceived to be a relatively simple tissue to replicate^7^.

Ultimately, cartilage consists of isolated chondrocytes within lacunae amidst extracellular matrix containing type II collagen, proteoglycans, elastic fibres, and other proteins that satisfy its structural and functional role. Articular cartilage is recognised to have a well-defined zonal organization where extracellular matrix and cellular organisation varies with tissue depth and this has been shown to affect its physical properties^8–12^. Nasoseptal cartilage has generally been assumed to be isotropic^13,14^ where studies investigating tensile properties and magnetic resonance spectroscopy, found no significant differences with respect to axis of testing i.e. directionally independent^15–17^.

However, malrotation of surgical cartilage grafts has been reported to lead to graft absorption, using both articular as well as nasoseptal samples^18–21^. These findings were attributed to collagen misalignment between graft and host, thereby suggesting that collagen orientation affected the strength of graft, raising the potential for anisotropy in nasoseptal cartilage^18–20^. Another study found a significantly higher compressive stiffness in the vertical/caudal-cephalic orientation compared to medial orientation of human nasoseptal cartilage^22^. Further work using microscopic magnetic resonance imaging and polarized light found a correlation between collagen orientation and compression stiffness, hinting at the importance of anisotropy for native tissue biomechanics^23^. However, there have been no further investigations elucidating the morphology of nasoseptal cartilage to explain these biomechanical findings.

Moreover, although aging has been shown to affect mechanical and biochemical properties of human nasoseptal cartilage^24,25^, no studies have investigated the effect of growth and development, and in particular, the time point at which anisotropy develops in nasoseptal cartilage i.e. pre-natal or post-natal. Lessons from articular cartilage have shown that structural changes occur in response to biomechanical stress postnatally^26^, reducing the thickness and increasing the matrix to cell ratio^27^. The theory is that resorption from the lower zones and appositional growth from the surface^28^ leads to zonal stratification and reorganization of collagen network^26,29^, resulting in distinct heterogeneity in functional characteristics^30^. The timeframe in which these changes occur varies between species; as fast as 3–4 months for lapine, 18 months for equine and 21 months for porcine cartilage^26,30–32^.

Much like misaligned cartilage grafts^21^, tissue engineered neocartilage has also experienced problematic reabsorption rates in the literature^33,34^. The reasons for this are likely to be multifactorial, but the importance of anisotropy for engineering durable tissue cannot be overlooked^35^, since it is recognised to affect biomechanical strength of tissues^18–20^. Engineered neocartilage often consists of immature, isotropic and homogenous tissue, which is unable to meet the functional and mechanical demands in the native environment^36^. The aim of this study was to compare the morphology and biomechanics of immature and mature bovine nasoseptal cartilage to determine levels of tissue tropism and whether this is a prenatal or postnatal phenomenon. Based on studies in articular cartilage, we hypothesized that the extracellular matrix content and structure would have an impact on the mechanical properties of mature nasoseptal cartilage. This interplay is crucial for understanding native cartilage development and may inform strategies for tissue engineering durable constructs.

## Results

### Morphological differences between immature and mature nasoseptal cartilage

**Figure 1:**
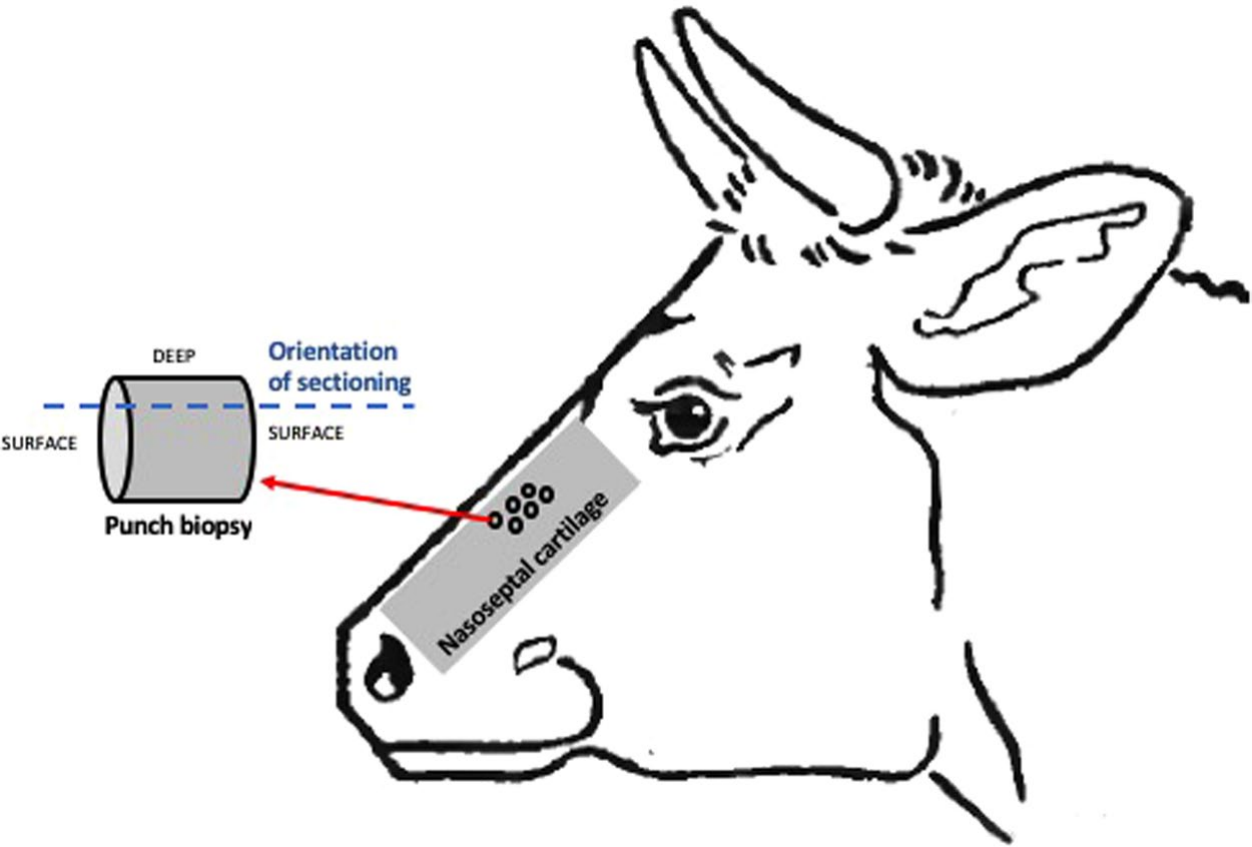
Bovine nasoseptal cartilage harvest. Schematic diagram to demonstrate orientation of bovine nasal cartilage biopsies and orientation of sectioning used for histology.

Nasoseptal cartilage from immature (1–3 week old) calves were 2.3 fold shorter (measured as the distance between nasal osteochondral junction proximally and membranous nasal septum distally) compared to that from mature adults aged 18–24 months (13.5 ± 1.2 cm versus 33.0 ± 3.0; p < 0.0001). Punch biopsies were taken from the central nasal septum and sectioned for histological staining in the orientation demonstrated in Fig. 1.

**Figure 2:**
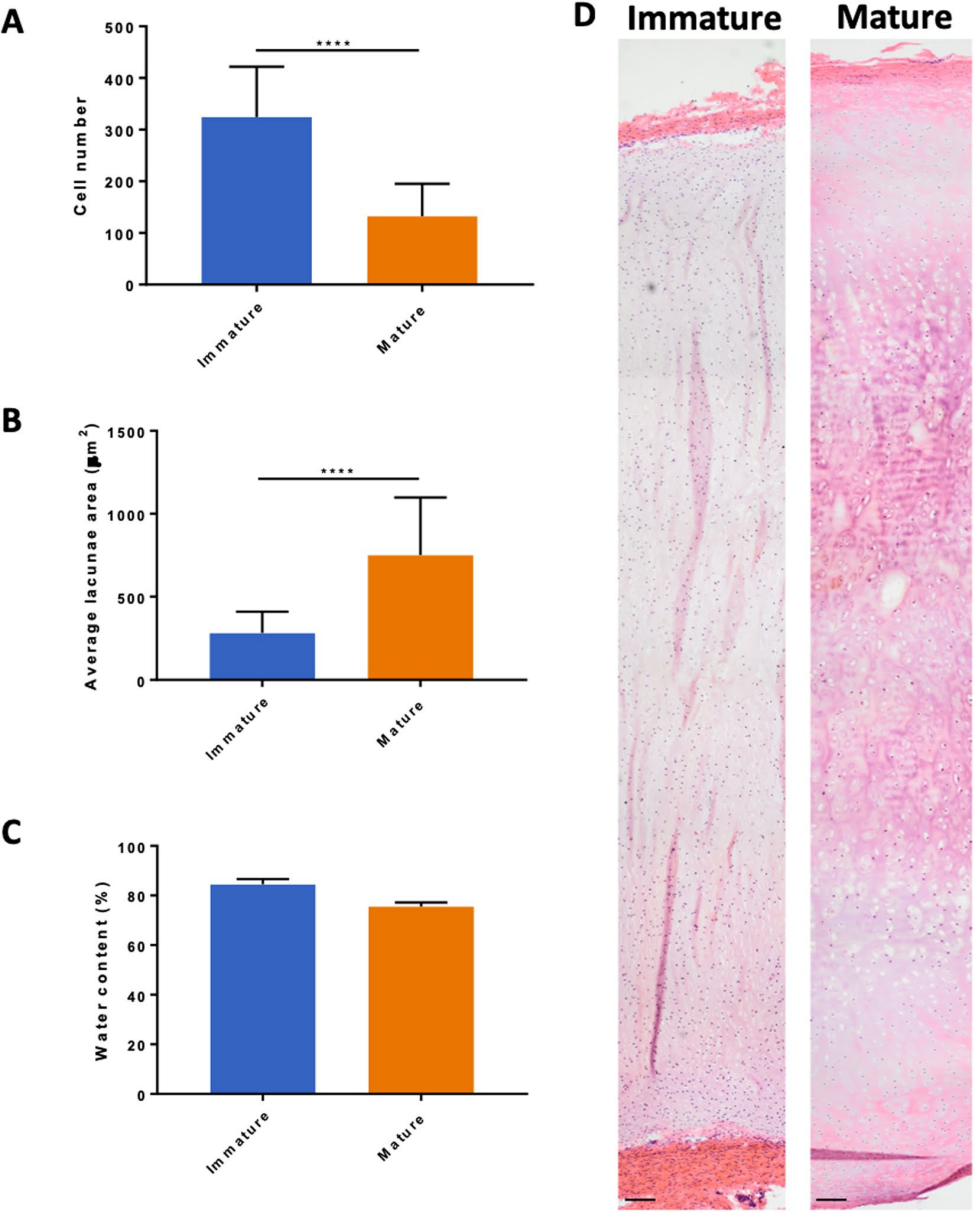
Overall morphological differences between immature and mature bovine nasoseptal cartilage. Cell number (**A**), lacunae size (**B**) and water content (**C**) of immature and mature nasoseptal cartilage. Data is expressed as the mean ± SD. Cell number was calculated over 5 areas in 3 biological replicates (n = 15). Lacunae size were determined from 20 measurements in 3 biological replicates (n = 60). Water content was determined for 3 repeats in 3 biological replicates (n = 9). Statistical differences were calculated using unpaired students T-test for cell number and lacunae area and Mann Whitney U test for non-parametric water content data. ****p < 0.0001. H&E histological stained sections (**D**) reconstructed using DoubleTake v2.5.1 software (Frederikssund, Denmark). Scale bar = 50 *μ*m.

Overall, immature bovine nasoseptal cartilage was found to have 2.4-fold greater cellularity (p < 0.0001) with smaller lacunae area (283 ± 126 μm versus 750 ± 348 μm; p < 0.0001) compared to mature cartilage (Fig. 2A,B). Although the percentage water content was greater in immature cartilage (85% ± 2.1 versus 76% ± 1.7; p = 0.0667) this difference was not statistically significant (Fig. 2C). The full depth of immature and mature nasoseptal cartilage was reconstructed using H&E stained sections to provide an overview of the morphological differences (Fig. 2D). Both mature and immature nasoseptum has an intensely eosin stained perichondral layer but immature cartilage appears more homogenous and cellular, whereas mature cartilage is more basophilic and hypertrophic in overall appearance (Fig. 2D). Mature cartilage was observed to decrease in cellularity with increasing tissue depth, with the cell morphology changing from ellipsoid in the surface layers to spheroidal in the deeper layers suggesting zonal stratification and anisotropy (Fig. 2D).

**Figure 3:**
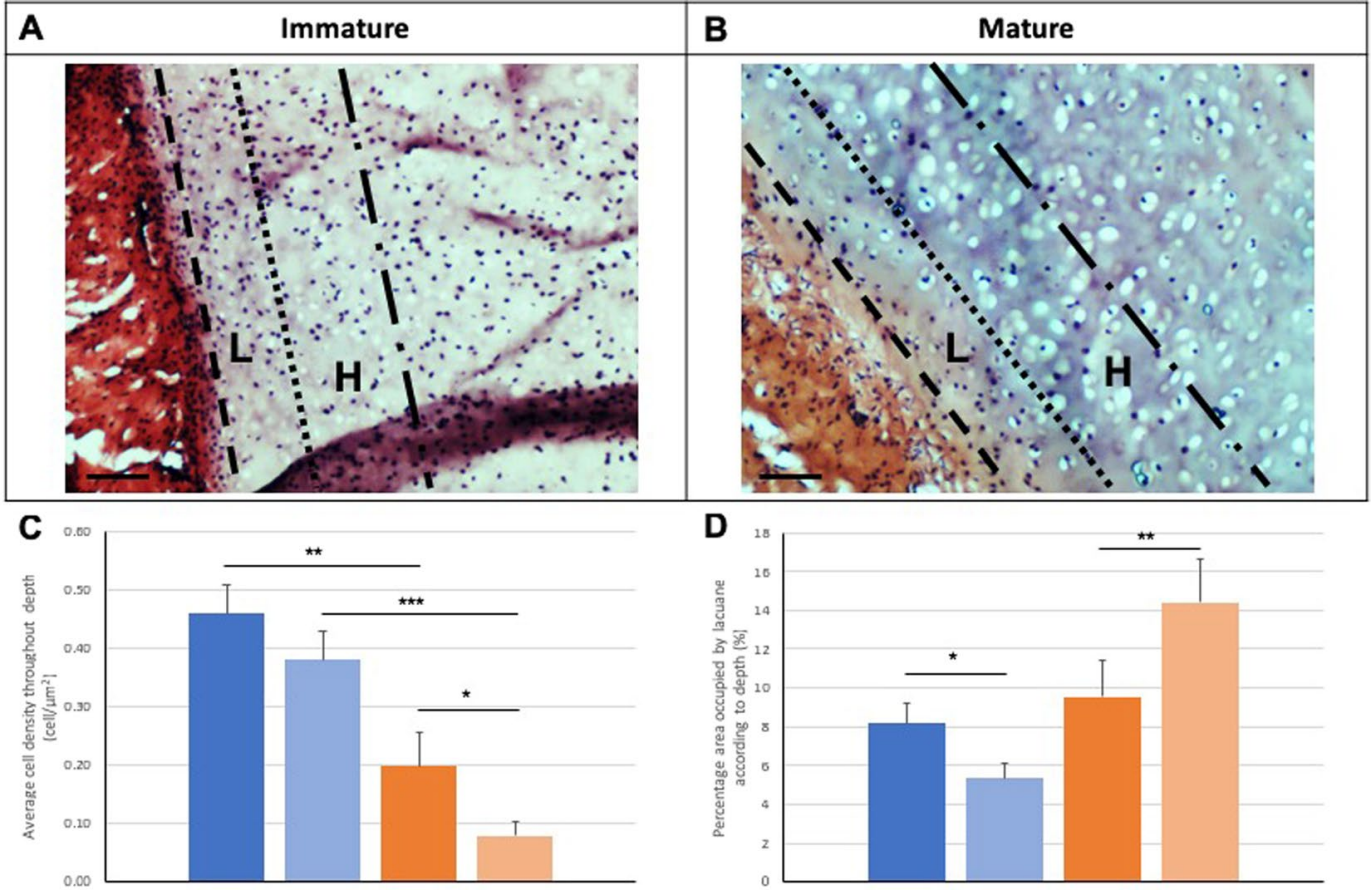
Zonal analysis of immature and mature nasoseptal cartilage. Sections were stained with haemotoxylin and eosin (**A**,**B**) to demonstrate overall morphology. Cellular density (**C**) and lacunae size (**D**) were calculated for immature (blue) and mature (orange) nasoseptal cartilage samples at low depth (L) (dark blue and dark orange) and higher depth (H) (light blue and light orange) of tissue. Data from 3 images for mature and immature, 20 measurements per image (n = 60). Data is expressed as the mean ± SD. Statistical differences calculated using one-way ANOVA and post-hoc analysis using Tukey’s HSD test. *p < 0.05, **p < 0.01, ***p < 0.001.

The morphological observations were confirmed with cell density measurements, which demonstrated a significant reduction from the low depth (L) to the high depth (H) zones of mature cartilage (0.20 ± 0.03 versus 0.08 ± 0.01; p = 0.03), unlike immature cartilage, that showed no significant difference (p = 0.118) (Fig. 3C). The area of lacunae increased with increasing depth in mature nasoseptal cartilage (596.85 ± 48.24 versus 818.95 ± 52.61; p = 0.003), whereas it decreased in immature cartilage (291.23 ± 17.42 versus 237.58 ± 17.06; p = 0.031) (Fig. 3D).

### Gene expression of immature and mature chondrocytes

**Figure 4:**
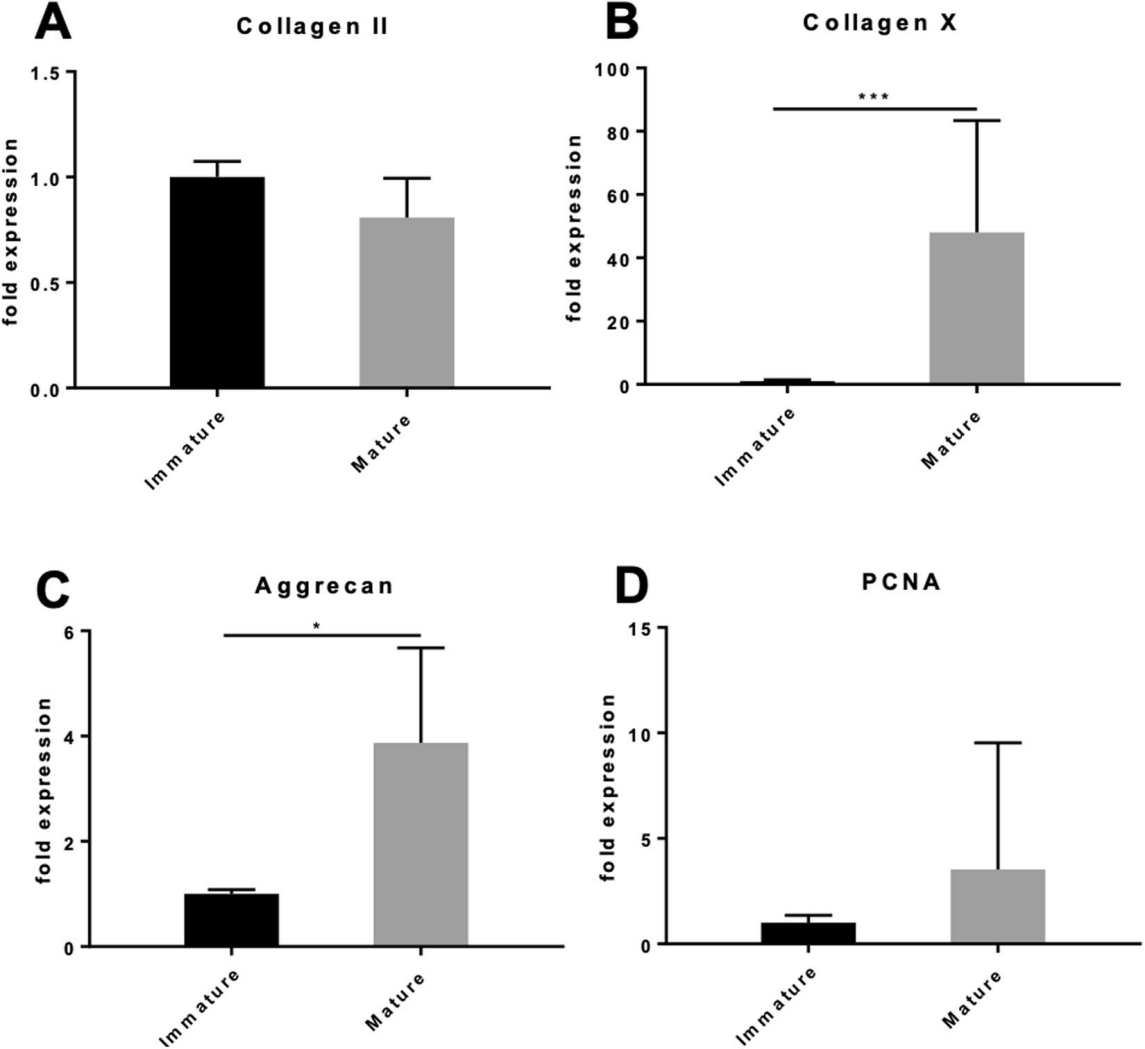
Relative gene expression and proliferation of immature and mature nasoseptal chondrocytes. The data shown is the ratio of the concentration of the genes of interest type II collagen (**A**), type X collagen (**B**), aggrecan (**C**) and PCNA (**D**) to 18 S ribosomal RNA (rRNA) housekeeping gene. Data is expressed as the mean ± SD, where the immature cartilage group was used as the calibrator group (normalized to expression level 1) (n = 4). Data is expressed as mean ± SD (n = 7). Statistical differences were calculated using unpaired students T-test. *p < 0.05, **p < 0.01, ***p < 0.001.

Gene expression analyses of type II collagen transcripts showed 19% decrease in relative expression in mature chondrocytes, but this was not statistically significant (p = 0.093) (Fig. 4A). Expression of type X collagen on the other hand, increased by 48-fold in mature compared to immature chondrocytes (p < 0.001) indicating a change to hypertrophic phenotype with maturation (Fig. 4B). There was also a 3.9-fold significant increase in expression of aggrecan in mature compared to immature chondrocytes (p = 0.02) (Fig. 4C). Mature chondrocytes demonstrated a 29% increase in relative expression of proliferating cell nuclear antigen (PCNA) compared to immature cartilage (Fig. 4D). PCNA gene expression differences were not statistically significant (p = 0.205).

### Extracellular matrix organization in immature and mature nasoseptal cartilage

**Figure 5:**
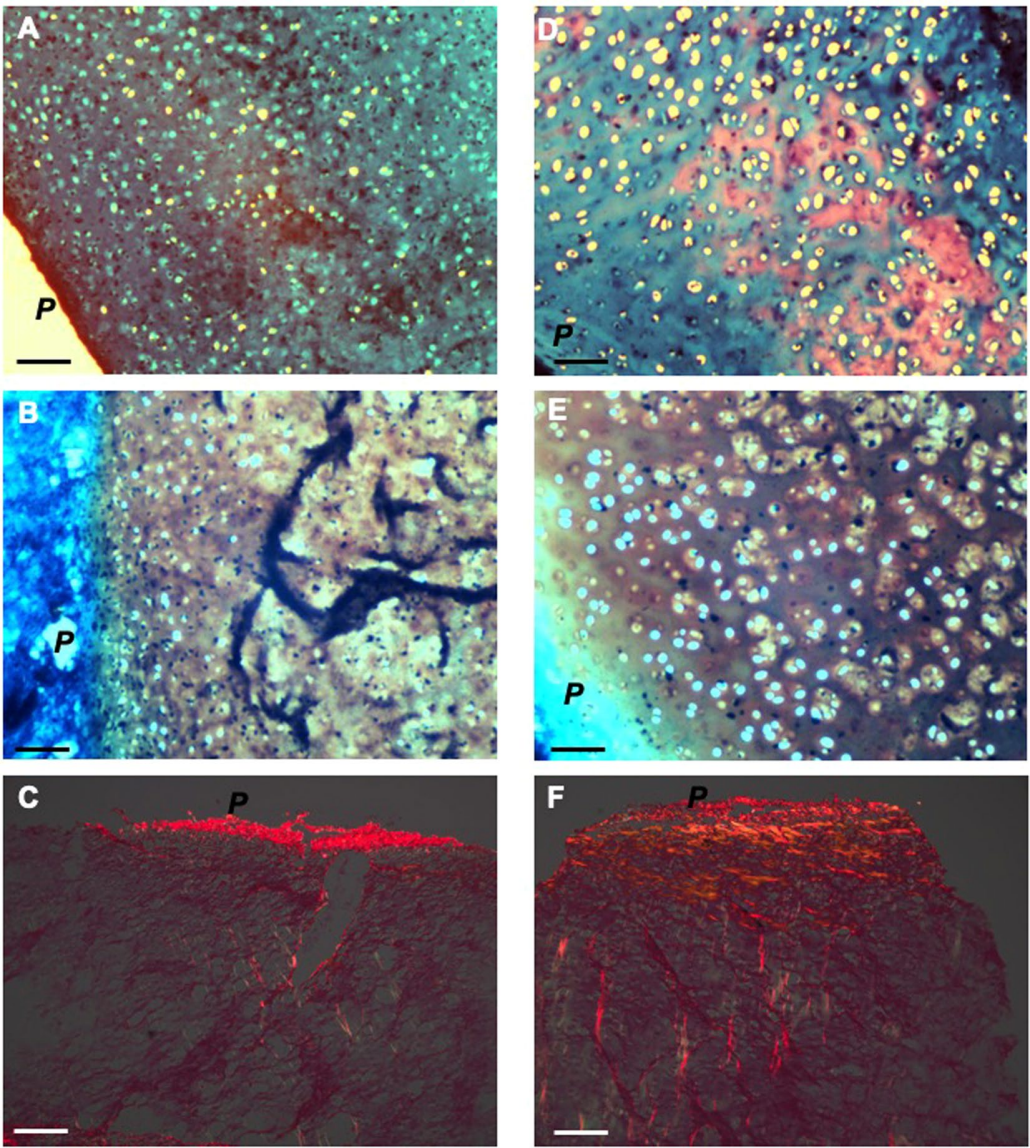
Histological sections of immature and mature nasoseptal cartilage. Immature (**A**–**C**) and mature (**D**–**F**) ections were stained with alcian blue (**A**,**D**), safranin-O (**B**,**E**) and pico-sirius red (**C**,**F**). *P* indicates perichondrial surface. Scale bar 30 μm.

**Figure 6:**
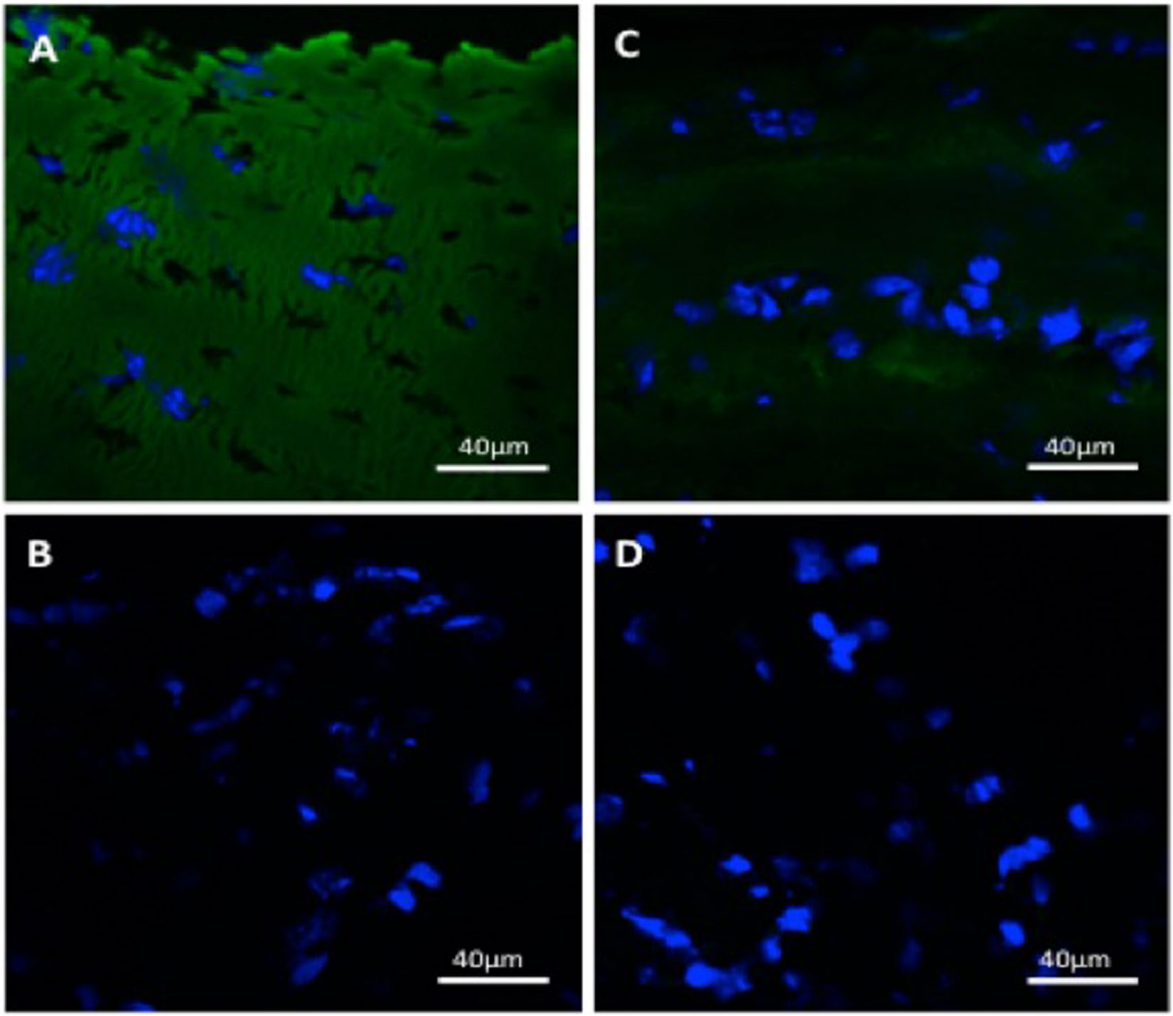
Immunohistochemistry for collagen type II in nasoseptal cartilage. Primary antibody collagen type II staining in immature (**A**) and mature (**C**) nasoseptal cartilage and their respective negative controls (**B**,**D**). Scale bar 40 μm.

In order to analyse extracellular matrix organization we assessed glycosaminoglycan content using alcian blue and safranin-O histological staining (Fig. 5) as well as collagen content and orientation using picro-sirius red visualized under polarized light microscopy (Fig. 5) and type II collagen immunohistochemistry (Fig. 6). Alcian blue staining demonstrated sulphated proteoglycans throughout immature and mature nasoseptal cartilage (Fig. 5A,D). Safranin-O staining shows orange-red positivity in immature cartilage (Fig. 5B) but appears more intense deep red throughout tissue depth in mature cartilage, suggesting increased glycosaminoglycan content (Fig. 5E). Picro-sirius red staining was strongest for collagen fibres on the cartilage surface that form the interface between perichondrium and chondrium, particularly evident for mature cartilage, demonstrating parallel alignment (Fig. 5F). At the high depth range immature cartilage again demonstrated a weaker signal overall, with collagen fibres observed to have an oblique arrangement (Fig. 5C) in contrast to the perpendicular alignment of mature cartilage (Fig. 5F).

Immunohistochemistry demonstrates a layered, non-homogenous distribution of collagen type II in mature nasoseptal cartilage versus the homogenous and more intense staining in immature cartilage (Fig. 6). The DAPI nuclei staining (Fig. 6) also supports the zonal stratification of cells in nasoseptal cartilage that is observed histologically (Fig. 2).

### Collagen fibre network and biomechanical characteristics of immature and mature nasoseptal cartilage

**Figure 7:**
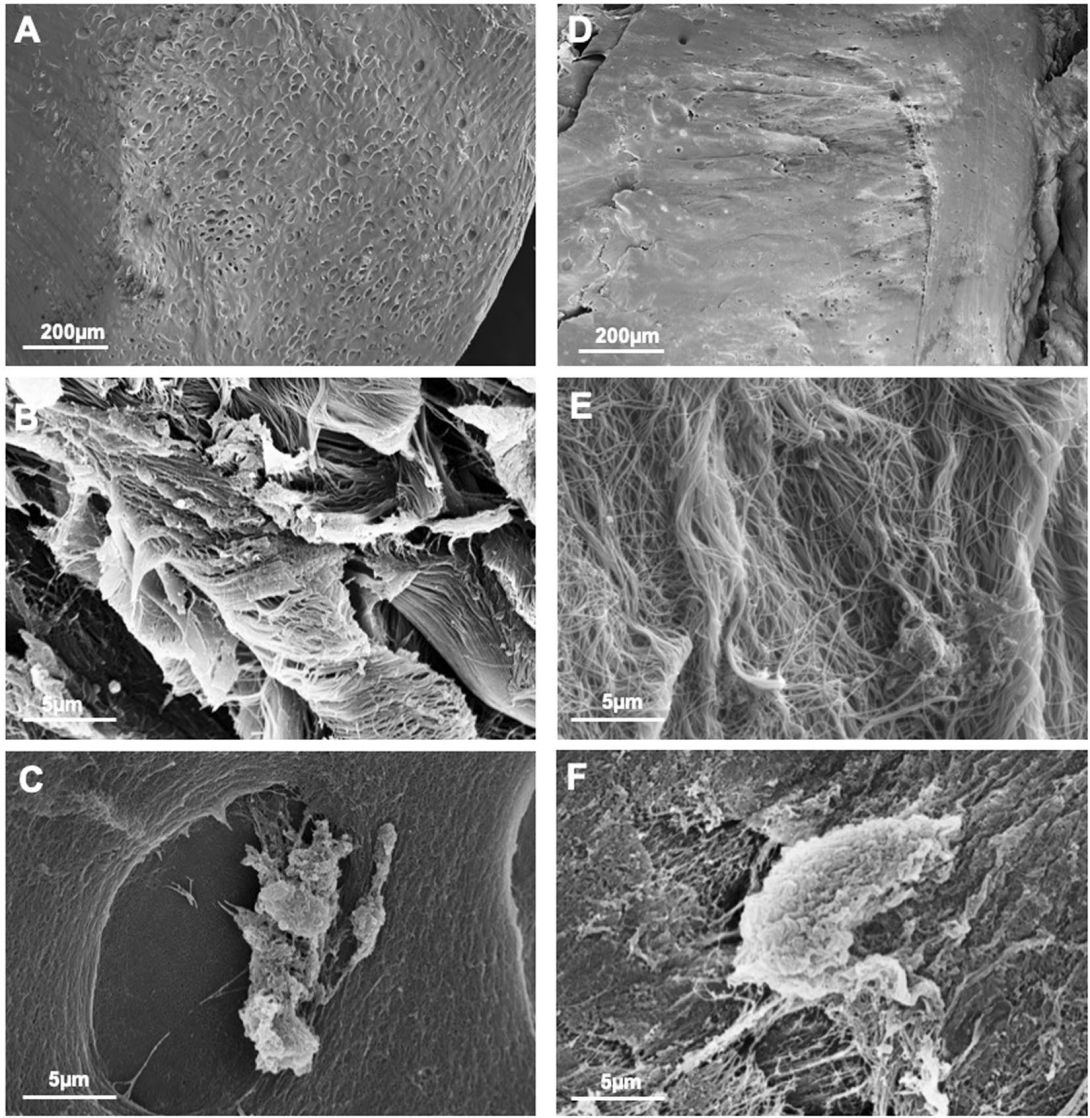
Scanning electron microscopy immature (**A**–**C**) and mature nasoseptal cartilage (**D**–**F**).

SEM analysis revealed that the superficial layer of mature nasoseptal cartilage contains collagen fibres which are finer, running in parallel to the surface and at higher density (Fig. 7E) compared to immature fibres, which are organized more randomly and at lower density (Fig. 7B), corroborating the picro-sirius red histology and immunohistochemistry findings shown in Figs 5 and 6 respectively.

**Figure 8:**
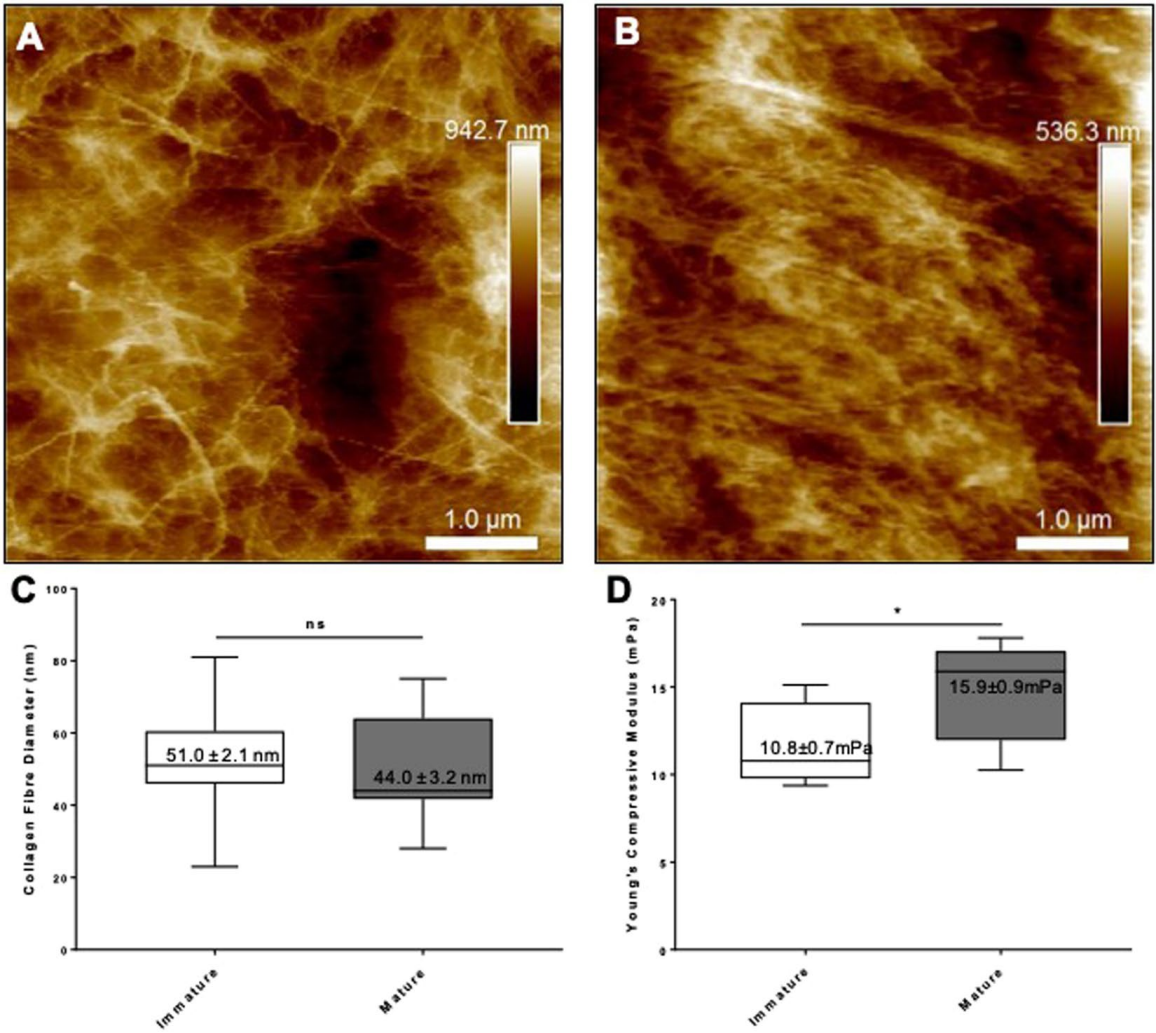
AFM and biomechanics of immature and mature nasoseptal cartilage. Topographical analysis of the surface profile of immature (**A**) and mature (**B**) nasoseptal cartilage. Box plots to demonstrate collagen fibre diameter (n = 15) (**C**) and Young’s compressive modulus (n = 10) (**D**). Data is expressed as the mean ± SD. Statistical differences were calculated using unpaired students T-test. ns = not significant. *p < 0.05.

Nanoscale topographical characterization using AFM confirmed that immature cartilage has a reticular and multidirectional organization of collagen fibres (Fig. 8A) compared to the more aligned collagen fibres of mature nasoseptal cartilage with higher density (Fig. 8B), providing further support for differences in collagen fibre orientation during maturation. Despite differences in collagen fibre orientation, no statistically significant differences in collagen fibre diameter were observed between immature (51 ± 2 nm) and mature (44 ± 3 nm) nasoseptal cartilage samples (p = 0.587) (Fig. 8C). Interestingly, the denser and more unidirectional collagen orientated mature cartilage was also demonstrated to have a significantly greater Young’s Compressive Modulus compare to immature nasoseptal cartilage (14.8 ± 2.8 mPa versus 11.5 ± 2.2 mPa; p = 0.0135) (Fig. 8D).

## Discussion

This study demonstrates that significant cellular, molecular, morphological and biomechanical differences exist between immature and mature bovine nasoseptal cartilage, suggesting a role for postnatal functional adaptation, a phenomenon previously reported in articular cartilage^30^. Immature nasoseptal cartilage was 2.4-fold more cellular (p < 0.0001) with smaller lacunae (p < 0.0001) and a homogenous appearance compared to mature cartilage, supported by the lack of differences in cellularity between the high depth and low depth regions (p = 0.118). Mature cartilage demonstrated anisotropic arrangement of cells, which reduced in density with increasing depth of tissue (p < 0.05) as well as lacunae, which increased with increasing depth (p < 0.01). Immunofluorescence findings also suggest a potentially zonal organization of cells and type II collagen extracellular matrix, which have a layered appearance in mature nasoseptal cartilage compared to the homogenous distribution of cells and collagen type II in immature samples. These findings suggest that changes in anisotropy in bovine nasoseptal cartilage occur postnatally in keeping with previous findings for articular cartilage^26,30–32^

Physical properties of cartilage depend greatly on both the content and structural organization of the extracellular components (collagen and proteoglycans)^22,23,32,37^. This study demonstrated that mature anisotropic nasoseptal cartilage had a significantly greater compressive stiffness compared to the more homogenous immature nasoseptal cartilage (p = 00135). This may be explained by the 3.9-fold greater aggrecan gene expression (p = 0.02) and safranin-O staining, indicating greater glycosaminoglycan content in mature nasoseptal cartilage. Although there were no significant differences in type II collagen gene expression between immature and mature cartilage, it was shown to be more homogenously distributed throughout immature cartilage at the protein level indicating regulation at translational rather than transcript level. Polarizing light microscopy of picro-sirius red stained sections revealed collagen fibrils are orientated parallel to the cartilage surface superficially and change to a perpendicular orientation with increasing depth in mature nasoseptal cartilage in keeping with the classical Benninghoff model of articular cartilage^38^. Immature cartilage birefringence was weaker, both with less parallel collagen fibres at the cartilage surface and less fibres with an oblique rather than perpendicular arrangement at higher depth. Postnatal remodelling of the collagen fibril architecture is recognised to have important biomechanical implications^31,32,39^. Perpendicular/vertical deep fibres in particular, are recognised to play a crucial role in increasing stiffness^40^ and may contribute the biomechanical differences observed between immature and mature nasoseptal cartilage in our study.

Post-natal changes in articular cartilage anisotropy have been attributed to mechanical joint load, which affects the metabolic activity of chondrocytes, thereby increasing proteoglycan synthesis and affecting collagen network remodeling, in order to withstand that load^26,41^. Although nasoseptal cartilage has no immediately obvious external loading force, as in all biological tissues and organs, there exists internal surface tension or tensegrity^42,43^. At the macroscopic level, this tensegrity results from the surface tension generated from the surrounding perichondrium, fibrous tissue and skin, which would be expected to increase as the tissue grows. In our study, immature bovine nasoseptal cartilage demonstrated 2.3-fold growth over 18–24 months (p < 0.0001). The decrease in cellularity and increase in distance between adjacent lacunae between immature and mature nasoseptal cartilage suggests this growth is due to extracellular matrix secretion and expansion rather than cellular proliferation. This is further supported by 1.8-fold lower *in vitro* proliferative rates in immature compared to mature chondrocytes (p = 0.0018). The secreted neomatrix in immature nasoseptal cartilage would therefore be expected to be initially isotropic. As the nasal septum grows, increased surface tension from the surrounding tissues as well as newly secreted matrix would be expected to further modulate the metabolic activity of chondrocytes in attempt to counteract these forces^41,43,44^.

## Conclusion

Our findings indicate that there is extensive postnatal synthesis and reorganization of the extracellular matrix in bovine nasoseptal cartilage. Mature nasoseptal cartilage exhibited both greater glycosaminoglycan content and collagen fibre network remodeling compared to immature cartilage and these changes correlate with biomechanical properties of nasoseptal cartilage. Interestingly, this remodelling in nasoseptal cartilage occurs independently of joint loading forces that have been attributed to be responsible for articular cartilage maturation, but the underlying molecular mechanisms remain to be elucidated. This highlights the potential importance of internal tissue tension forces in generating tissue anisotropy with implications in designing the optimal microenvironment for cartilage tissue engineering.

## Materials and Methods

### Nasoseptal cartilage harvest

Bovine nasoseptal cartilage was harvested from calves (1–3-week-old) and mature adults (18–24-month-old) on the day of slaughter from Cig Calon Cymru Cyf Abattoir (Heol Parc Mawr, Cross Hands, Llanelli, Dyfed) following Food Standards Agency (FSA) approval. Nasoseptal cartilage was isolated by cutting though the naso-frontal groove and washed and sterilised using 70% ethanol (Fig. 1A). The mucosa was dissected away under sterile conditions using a surgical blade to expose the underlying nasoseptal cartilage. Full depth cartilage biopsies (6 mm) were taken from the central nasoseptal cartilage and flash-frozen in n-hexane in a dry-ice bath and stored as explants in −80 °C until scanning electron microscopy analysis or immunohistochemistry analysis or fixed in 10% formalin for 24 hours and stored in PBS until paraffin embedding for histological analysis. Freshly dissected immature cartilage explants from immature and mature animals (n = 3 per group) were weighed, then flash-frozen in n-hexane, and lyophilized by freeze-drying for 12 hrs. Dried samples were reweighed to determine the percentage water content of samples.

### Histology

Bovine nasoseptal cartilage biopsies were embedded in optimal cutting temperature compound (OCT, VWR, Radnor, PA, USA) and sectioned to 8 μm using a cryotome (following orientation in Fig. 1B). Sections were stained for nuclei and cytoplasmic labelling using haematoxylin and eosin (H&E) staining. All stains were visualized using phase-contrast AmScope MD35 (AmScope Irvine, CA, USA). To allow objective assessment of cellularity and lacunae area the H&E stained sections were divided according to low depth (L) (defined as immediately below the perichondrium to 30 μm in depth) and high depth (H) (adjacently deep to low depth region at 50 μm in depth) regions and analysed using Image J (National Institute of Health). Sections were stained with 1% alcian blue (TCS Biosciences Buckingham, UK) solution for 15 minutes. Safranin-O staining was performed using 0.1% fast green (TCS Biosciences) solution for 10 minutes followed by immersion in 1% acetic acid for 10 seconds (Sigma-Aldrich, St. Louis, MO, USA) and staining with 0.1% Safranin-O (TCS Biosciences) for 20 minutes.

For picro-sirius staining to detect collagen orientations, paraffin embedded nasoseptal cartilage biopsies were sectioned using a microtome at 10 μm and mounted on poly-L-lysine coated slides according to orientation described in Fig. 1B. Sections were dewaxed at 45 °C overnight and hydrated by treatment with a series of washes (histochoice three times for 5 minutes followed by 100% ethanol twice for 2 minutes, then 95% ethanol for 2 minutes, 70% ethanol for 2 minutes, distilled water for 2 minutes) and air dried. Slides were stained with 1% picro-sirius red, dehydrated, mounted in DPX mountant (Cellpath, Newtown, UK) and imaged using a phase-contrast AmScope MD35 (AmScope) light microscope and polarized light microscopy (DM4 P, Leica, Germany) was used to detect birefringence.

### Immunohistochemistry

Fresh frozen nasoseptal cartilage biopsies were cryosectioned at 10 μm thickness using a cryostat microtome (Leica Biosystems, Wetzlar, Germany) and stored at −20 °C prior to staining. At room temperature sections were rehydrated with distilled water for 2 minutes. Ice cold 95% ethanol was added for under 1 minute, followed by washing twice with PBS/TBS buffer with 0.1% Tween 20 (PBST/TBST) for 5 minutes and drawing around the sections with a Dako pen. 1 mg/ml of hyaluronidase and 0.1 U/ml of chondroitinase ABC (Ahrens 2011) in PBST/TBST were added to the sections for 1 hour at 37 °C, followed by washing twice with PBST/TBST for 5 minutes. 10% goat serum in PBST was used for blocking for 1 hour at room temperature prior to staining with type II collagen primary antibody (II-II6B3-s, 22 ug/ml) (DSHB): a mouse monoclonal antibody, 1:10 dilution) overnight at 4 °C. The sections were washed three times with PBST/TBST for 5 minutes prior to staining with secondary antibody goat anti-mouse IgG-FITC (Santa Cruz, sc-2010): 1:100 before washing for a further three times and adding SlowFade® Gold Antifade Mountant with DAPI (Thermofisher Waltham, MA, USA) and mounting coverslip. Images were taken using confocal microscopy (Zeiss Laser Scanning Microscope 510). Cellular density was determined by counting DAPI stained nuclei for nuclei using Image J software and calculated as the total cells per unit area.

### Reverse transcription-quantitative polymerase chain reaction (qRT-PCR) analysis of gene expression

Immature and mature nasoseptal cartilage samples were first flash frozen in n-hexane (Sigma-Aldrich) in a dry-ice bath and then homogenized using a Mikro-Dismembrator U (B. Braun Biotech International, Allentown, Pennsylvania, USA) in the presence of 250 μl TRI Reagent (Applied Biosystems, Foster City, USA) for 2000 rpm 1 min for 2 cycles. The sample was thawed and then 0.2 ml chloroform (Sigma-Aldrich) added, vortexed and centrifugation to allow phase separation. An equal volume of 70% ethanol was added to the aqueous phase and the mixture added to a RNeasy mini-column (Qiagen, Hilden, Germany) and processed for RNA isolation using the manufacturers recommended protocol with a DNAse I digestion step and converted to complementary DNA (cDNA) using Superscript IV reverse transcriptase (Thermofisher Scientific) and random primers (Promega, Southampton, UK) following manufacturer’s protocol. QRT-PCR reactions were prepared in duplicates using GoTaq® qPCR Master Mix (Promega) 5 ng of cDNA, and 0.3 mM forward and reverse primers of COLII, ACAN, COLX, PCNA (Table 1). Primers were designed using Primer 3 and specificity confirmed by BLAST and plasmid DNA sequencing. Reactions were performed on a Stratagene Mx3000 real-time PCR analyser (Agilent Technologies, Edinburgh, UK). Standard curves over the linear range of amplification were generated for all primer sets, and data was used where the efficiency of amplification was between 90% and 105% and the melt curves generated a single product.

**Table 1:** Summary of primer sequences of the genes of interest.

Gene		Primer sequence	Tm (°C)	Annealing temperature
Bovine *RPS18*	Forward	CAC-TGG-AGG-CCT-ACA-CGC-CG	68	55
	Reverse	AGG-CAA-TTT-TCC-GCC-GCC-CA	64	
Bovine *COLII*	Forward	CT-GGA-TGC-CAT-GAA-GGT-TTT	60	55
	Reverse	GC-TCC-ACC-AGT-TCT-TCT-TGG	62	
Bovine *COLX*	Forward	CCC-ATG-CTT-GGG-TAG-GTC-TG	59	55
	Reverse	CCA-TAC-CTG-GTC-GTT-CTC-GG	60	
Bovine *LOXL1*	Forward	GCG-TTT-CCC-CCA-GCG-TGT-GA	66	55
	Reverse	GCT-GTG-GTA-GTG-CTG-GTG-GCA	53.1	
Bovine *PCNA*	Forward	GTG-AGG-AGT-CAA-CCA-AGA	62	55
	Reverse	GGA-TAC-AGT-GAG-TTC-TAC-CA	63	

RPS18, 18s ribosomal RNA; COLII, collagen type II; COLX, collagen type X; ACAN, aggrecan; PCNA, proliferating nuclear antigen.

### Scanning electron microscopy (SEM)

Defrosted bovine nasoseptal cartilage biopsies were washed three times with 50 mM Sodium Cacodylate-HCl Buffer solution (pH 7.2–7.4, SPI Supplies) at 10 to 20 minute intervals to remove excess salt. The samples were fixed overnight in 2% Glutaraldehyde (Sigma Aldrich, UK) and dehydrated with a series of graded concentrations (30% to 100%) of ethanol. The dehydrated sample was then rinsed with 50% Hexamethyldisilazane solution (HMDS) in 100% ethanol for 10 minutes in a fume hood and then three times in 100% HMDS and left overnight to dry. The sample was coated with a thin layer of gold (∼15 nm) using sputter coating and was imaged using scanning electron microscopy (Hitachi 4800).

### Atomic force microscopy (AFM)

Dissected nasoseptal cartilage explants, previously stored at −80 °C, were analysed in PBS buffer at 37 °C using a Bioscope Catalyst (Bruker Instruments, Santa Barbara, California, USA). Bruker MLCT silicon nitride cantilevers (20 nm tip radius) were calibrated on clean glass slides and used for force spectroscopy and sample imaging in PeakForce modality. For each sample, at least 5 different areas were analysed, where each area ranged from 25 to 400 μm^2^. Image offline processing included low-order flattening and plane fitting using Nanoscope Analysis, v.1.50. Fibril diameter was measured using ImageJ on AFM topographical data with a total of 15 fibrils per sample.

### Biomechanical testing

Unconfined compression testing was performed on nasoseptal cartilage cylinder discs (6 mm in diameter and approximately 3 mm in thickness) using the BOSE ElectroForce® 3200 mechanical loading machine (Bose Corporation, ElectroForce® Systems Group, Minnesota, USA). The compressive strength of the samples was tested at loading regime has to be mentioned here N and cycles at 1 Hz. The displacement and load were recorded, and Young’s modulus was determined as the slope of the linear region of the stress-strain curve.

### Isolation of bovine nasoseptal chondrocytes

Fresh bovine nasoseptal cartilage biopsies were placed into sterile DMEM (Invitrogen, UK) and minced it into 1 mm^3^ pieces prior to sequential pronase (Roche, 0.4%, 1 hour at 37 °C) and collagenase (Sigma, 0.2% for 10 hr at 37 °C^7^, digest with gentle agitation. The mixture was filtered through 40 μm cell strainer (VWR), centrifuged (500 rcf for 5 minutes) to remove enzyme mixture, re-suspended in culture media (containing DMEM supplemented with Penicillin 10000 mg/ml, Streptomycin 10000 U/ml, 0.1 mM ascorbic acid, 0.5 mg/ml L-glucose, 100 mM HEPES, 1 mM sodium pyruvate, 2 mM L-glutamine and 10% fetal bovine serum (FBS)) and seeded at 2700 cells/cm^2^ for cell expansion^45,46^. Once > 70% confluent, cells were incubated in 0.05% trypsin-EDTA (Thermofisher) for 5–10 minutes at 37 °C for further expansion.

### Statistical analysis

Sample values are shown as means ± standard deviation. Statistical analysis of multiple groups with normally distributed data was performed using one-way analysis of variation (ANOVA) with post-hoc analysis performed using the Tukey HSD test performed using Prism software (Graphpad Inc., La Jolla, USA). A p value of <0.05 was defined as the level of significance.
