# Author Correction: Morphological and biomechanical characterization of immature and mature nasoseptal cartilage

**DOI:** 10.1038/s41598-020-61552-8

**Published:** 2020-03-10

**Authors:** Zita M. Jessop, Yadan Zhang, Irina N. Simoes, Ayesha Al-Sabah, Nafiseh Badiei, Salvatore A. Gazze, Lewis Francis, Iain S. Whitaker

**Affiliations:** 1 Reconstructive Surgery and Regenerative Medicine Research Group, Swansea University Medical School, Swansea, UK; 2 The Welsh Centre for Burns and Plastic Surgery, Morriston Hospital, Swansea, UK; 3 Centre for NanoHealth, Institute of Life Sciences, Swansea University, Swansea, UK

Correction to: *Scientific Reports* 10.1038/s41598-019-48578-3, published online 28 August 2019

The original version of this Article contained a typographical error in the spelling of the author Irina N. Simoes, which was incorrectly given as Irina Simoes. This has now been corrected in the PDF and HTML versions of the Article.

